# Decoding the Effect of Age on the Taste Perception of Chicken Breast Soup Based on LC-QTOF-MS/MS Combined with a Chemometric Approach

**DOI:** 10.3390/foods12030674

**Published:** 2023-02-03

**Authors:** Lili Zhang, Li Liang, Kaina Qiao, Dandan Pu, Baoguo Sun, Xuewei Zhou, Yuyu Zhang

**Affiliations:** 1 Food Laboratory of Zhongyuan, Beijing Technology and Business University, Beijing 100048, China; 2 Key Laboratory of Flavor Science of China General Chamber of Commerce, Beijing Technology and Business University, Beijing 100048, China; 3 College of Food Science and Engineering, Tianjin University of Science and Technology, Tianjin 300457, China

**Keywords:** chicken soup, age, taste compounds, LC-QTOF-MS/MS, OPLS-DA

## Abstract

A nontargeted fingerprinting approach combined with the chemometrics method and sensory analysis was used to assess the differences in taste-chemical compositions of chicken breast soup with different ages and their sensory qualities. The sensory evaluation results showed that the overall taste as well as the sourness, saltiness, and umami scores of the soup were increased with the age of chicken. Fifty-nine compounds were identified from four soup samples by liquid chromatography-tandem quadrupole time-of-flight mass spectrometry (LC-QTOF-MS/MS), and their total content was the highest in the 90 wk soup samples. Six upregulated compounds (carnosine, hypoxanthine, inosine, inosine 5′-monophosphate (5′-IMP), adenosine 5′-monophosphate (5′-AMP), and lactic acid) were identified as potential contributors to the taste characteristics of the 90 wk soup samples by orthogonal projections to latent structures–discriminant analysis (OPLS-DA). Additional experiments showed that 5′-AMP particularly contributed to the sourness of the soup, while carnosine contributed to the saltiness and umami of the soup.

## 1. Introduction

Chicken soup is very popular for its delicious taste and rich nutrition. Studies have proven its immunity-regulating, fatigue-relieving, and memory-strengthening benefits [1]. Nucleotides, amino acids, organic acids, peptides, and their derivatives were considered to make an important contribution to the taste characteristics of chicken soup [2]. The age of chicken is one of the factors that affect the taste-chemical compositions of chicken soup. Some native chickens with a longer growing period (Hinai-jidori chickens of Japan, native chickens of Korean, Kadaknath chickens of India, etc.) have been shown to possess better taste qualities than broilers with a shorter growing period [3,4]. Lengkidworraphiphat et al. reported that the tasty amino acids (asparagine, threonine, serine, glutamic acid, glycine, and alanine) content in Thai native chickens (16 weeks old (wk)) were significantly higher than those in broilers (6 wk) [5]. Organic acids and small peptides were found to be the dominate compounds in Wuding chicken aged 15–32 wk, and the concentrations of lactic acid and creatine increased with the rearing times within 28 wk [6]. Jayasena et al. reported that the contents of inosine, linoleic acid, and lysine increased with the age of chicken (10–14 wk) in defatted freeze-dried chicken soup, while the contents of 5′-AMP, hypoxanthine, and oleic acid decreased [7]. The age of the chicken resulted in a different composition of taste compounds in meat, which affects the taste of soup. Therefore, clarifying the correlation between chicken age and taste quality of the soup is important for the development of chicken soup products.

Old hens are superior to young chickens in terms of taste presentation and aroma precursors, which make them the best choice for chicken soup [8]. Numerous prior studies have focused on the taste profiles of native chickens versus commercial broilers and have emphasized the taste superiorities of native chickens. However, the native chickens with long growing periods and high prices could not meet the needs of consumers [9]. Hens aged over 80 wk are one of the major byproducts of the laying hens industry and are possible alternatives for native chickens. Lakshani et al. reported that old hens exhibited higher contents of eicosapentaenoic acid and docosahexaenoic acid than commercial broilers [10]. The flavor-related amino acids (valine, isoleucine, leucine, phenylalanine, arginine, proline, and methionine) were shown to be at higher levels in old hens (72 wk) than Thai native chickens (16 wk) in a previous study [5]. Zhang et al. also proved that old hens (90 wk) are a natural source of umami peptides (PPQEAAQF, AEEHVEAVN, NEFGYSNR, etc.) [11]. A scientific analysis of the flavor qualities of old hens could effectively enhance their value and improve the economic benefits of the laying industry. However, little information is available on the taste properties of old hens and, in particular, the substances that prominently contribute to the taste profiles of old hens are not clear.

In this work, the differences in taste compounds and taste properties between soup samples prepared from four ages of chickens (10, 20, 60, and 90 wk) were investigated. The aims of this work were to (1) identify and quantify compounds in four soup samples based on a nontargeted analytical method; (2) investigate the correlations between the taste properties of the soup and the age of the chicken; and (3) excavate and verify the compounds with key contributions to the taste of chicken soup through orthogonal projections to latent structures–discriminant analysis (OPLS-DA) combined with additional experiments.

## 2. Materials and Methods

### 2.1. Materials and Reagents

Chicken breast (10, 20, 60, and 90 wk Hy-Line brown) was purchased from the family farm of the Zhongcui family of Daiyue District (Taian, China). The basic ingredients of the four chicken breast are shown in Table S1. Methanol, acetonitrile, and ammonium formate (all LC-MS grade) were purchased from Fisher Scientific (Shanghai, China). The ultrapure water was prepared with a Milli-Q water purification system (Millipore, Bedford, MA, USA). Ala-Phe (99.66%) was synthesized from Nanjing peptide Biotech Co., Ltd. (Nanjing, China). All other chemicals were analytical reagents, and their acquisition and purity information are shown in Table S2.

### 2.2. Preparation of Chicken Soup

Chicken breast soup was prepared according to our previous method [12]. The diced chicken breast (5 cm side length) was blanched for 1 min and placed in a stew pot. After adding water (1.5 times the meat weight), the breasts were stewed for 4 h to obtain the chicken breast soup.

### 2.3. Sensory Evaluation

A quantitative descriptive analysis (QDA) was used for sensory analysis according to the reported literature [13]. The sensory evaluation panel was composed of five females and five males (aged from 22 to 29). They were able to distinguish the basic tastes (bitterness, sweetness, sourness, saltiness, and umami) and had certain experiences in QDA analysis in meat products. Panelists were trained to distinguish and reorder the 5 basic taste solutions in different concentrations for 20 min three times per week (3 weeks). Four kinds of chicken soup were served to the subjects during training to provide a broad range of sensory variability for each attribute and further stimulate the formation of descriptors. The final evaluation criteria of sensory evaluation were determined by a panel discussion. When the sensory evaluation score of five basis tastes was set 5, the corresponding standard solutions were 0.05% citric acid (sourness), 2% sucrose (sweetness), 0.00075% quinine (bitterness), 0.3% sodium chloride (saltiness) and 0.08% monosodium glutamate (umami). The overall taste was used to evaluate the acceptability of the sample taste on a scale of 0–10.

Four chicken soup samples (10, 20, 60, and 90 wk) were cooled to 25 °C for sensory analysis. The experiments were repeated three times for each sample. All sensory tests of this work were approved by the Ethics Committee of China Agricultural University (CAUHR2021022, Beijing, China).

### 2.4. Identification of Compounds by LC-QTOF-MS/MS

The soup samples were placed in the refrigerator (4 °C) for 24 h and were centrifuged to remove the solidified fat (10,610× *g*, 4 °C, 15 min). Then, the supernatants were filtered (medium-speed qualitative filter paper, Φ 15 cm) and lyophilized (condensing temperature < −80 °C, vacuum value < 5 Pa) considering their inconvenient storage and perishable properties. Three milliliters of methanol aqueous solution (*V*:*V* = 1:1) were added to the lyophilized powder (0.1 g). Then, supernatant A was obtained after vortexing (1 min) and centrifugation (10,610× *g*, 4 °C, 10 min). The residue was added with 3 mL of a dichloromethane/methanol solution (*V*:*V* = 3:1), vortexed for 1 min, and then centrifuged (10,610× *g*, 4 °C, 10 min) to obtain supernatant B. Supernatant A and B were mixed evenly and then dried with nitrogen. Finally, 1 mL of a 20% acetonitrile aqueous solution was added for reconstituting. The samples were filtered through a 0.22 μm membrane before instrumental analysis.

The identification of the compound was performed using LC-QTOF-MS/MS according to the method of Huang et al. with minor modifications [14]. A Nexera X2 UHPLC LC-30AD system (Shimadzu, Kyoto, Japan) coupled to a quadrupole time-of-flight mass spectrometer (X500R QTOF, SCIEX) with an electrospray ionization source (ESI) was used in this work. A dynamic background subtraction (DBS) trigger information-dependent acquisition (IDA) mode was used for data acquisition. The sample was separated on a Kinetex^®^ 2.6 μm EVO C18 column (100 × 4.6 mm). The mobile phase consisted of A (0.1% formic acid in water) and B (0.1% formic acid in acetonitrile) for the ESI^+^ mode, while the mobile phase consisted of C (5 mM ammonium formate in water) and D (90% acetonitrile/water solution with 5 mM ammonium formate) for the ESI^−^ mode. A gradient elution was carried out according to Table S3. The flow rate, injection volume, and column temperature were set at 0.6 mL/min, 10 μL, and 40 °C for both modes, respectively. The key parameters of the mass spectrometer in ESI^+^ and ESI^−^ mode were carried out according to Table S3. Quality control (QC) samples were inserted every five samples in the run sequence to monitor the precision of the analytical process. The identification of the compounds in the samples was based on the high-resolution database provided by SCIEX and the database established by the laboratory and was ensured by the standard. Finally, each compound was quantified by the external standard method. Each sample was repeated ten times.

### 2.5. Addition Experiments

The differential compounds of three sample groups (10/90, 20/90, 60/90 wk) were identified with OPLS-DA. The additional experiments were designed according to the content difference of each differential compound. The taste characteristics of the differential compounds and chicken soup supplemented with differential compounds were analyzed with the electronic tongue to verify the taste-improving effect of the differential compounds on chicken soup. The concentration of each differential compound to be added to the chicken soup was calculated by subtracting the concentration of the differential compound in the 10, 20, or 60 wk soup samples from the concentration in the 90 wk sample.

### 2.6. Electronic Tongue Determination

The analysis method of the electronic tongue was performed according to Liang et al. [15]. The SA402B taste sensor system, with three sensor probes (AAE for umami taste, CT0 for saltiness, CA0 for sourness) and two reference probes was used for the analysis of the taste characteristics of the samples. A KCl solution (10 mM) was used as a solvent instead of ultrapure water to determine the taste characteristics of differential compounds to reduce the error in the data transformation.

### 2.7. Statistical Analysis

The analysis of HCA, PCA, and OPLS-DA was performed with the SIMCA14.1 software package (V14.1, MKS Data Analytics Solutions, Umea, Sweden). Statistical analysis was carried out using the Kruskal–Wallis test via SPSS version 25.0 software (IBM Corp., Armonk, NY, USA). *p* values were corrected for multiple testing using the Benjamini–Hochberg method. The taste analysis system application (Insent, Japan) was used to process the electronic tongue data.

## 3. Results and Discussion

### 3.1. Taste Properties of Four Soup Samples

**Figure 1:**
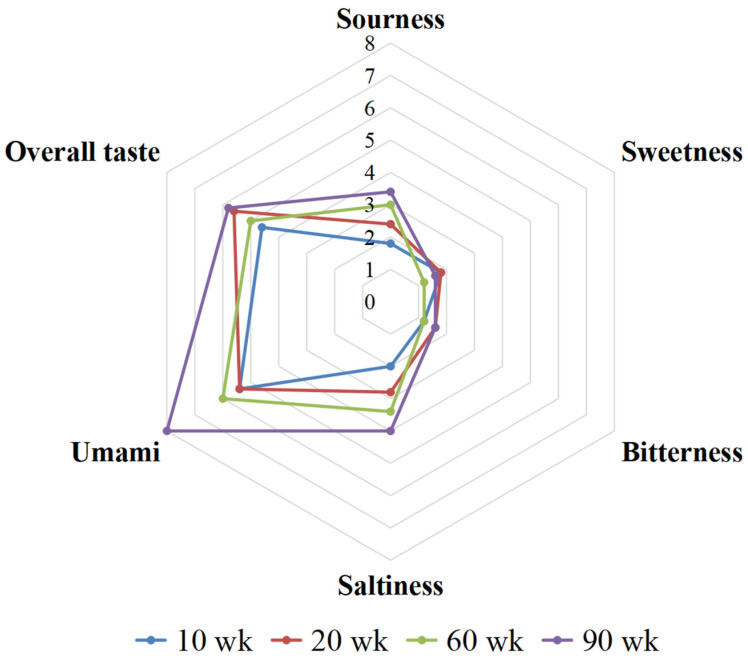
Taste profiles of four chicken soup samples.

The sensory evaluation results of the 10, 20, 60, and 90 wk soup samples are shown in Figure 1 and Table S4. It can be seen that the four soup samples were mainly tasted as umami, salty, and sour, with umami being the strongest. This result was confirmed by our previous study that chicken breast soup was dominated by an umami taste [12]. The higher accumulation of 5′-IMP in Type IIB muscle fibers and its synergistic effect of free amino acids might be one of the reasons for the prominent umami taste of breast meat [16]. As shown in Table S4, the sweetness and bitterness of the four soup samples were low intensity and had no significant effect at the different ages of the chicken. The scores of sourness and saltiness increased with chicken age, with the highest scores for both being observed in the 90 wk soup samples. The umami and overall taste of the four soup samples were generally positively correlated with the age of the chicken. Notably, the umami intensity of the 90 wk soup was significantly higher than that of the 10 and 20 wk soup samples, which might be the reason that consumers preferred to choose aged chicken to prepare chicken soup. The umami and saltiness were also considered as the dominant taste of Sanhuang old hen (Chinese native chicken) soup in a previous study by Liang et al. [15].

### 3.2. Compounds Identified in Four Soup Samples

**Figure 2:**
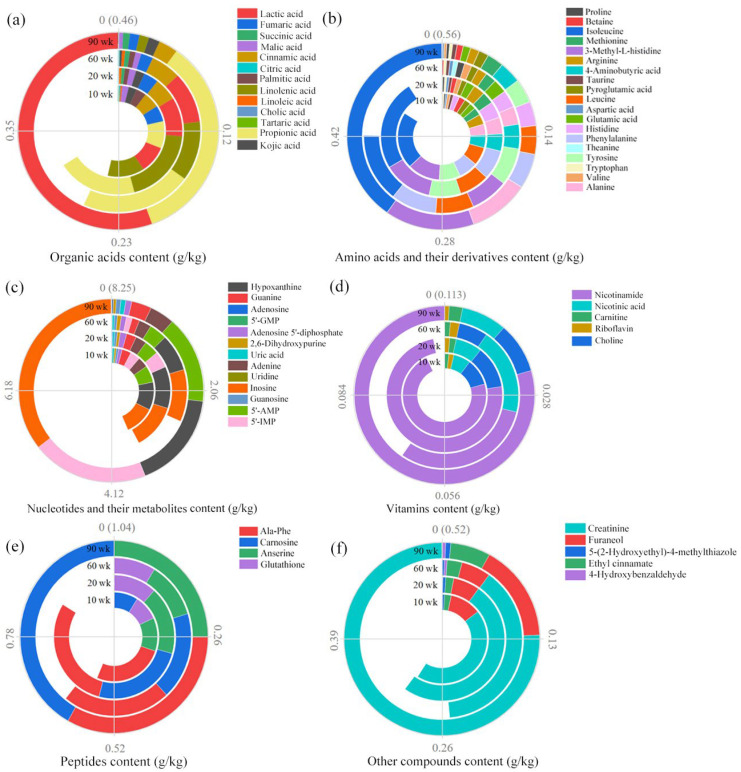
Quantitative results of six types of compounds (organic acids (**a**), amino acids and their derivatives (**b**), nucleotides and their metabolites (**c**), vitamins (**d**), peptides (**e**), other compounds (**f**)).

The identification results of the compounds are shown in Table S5. Fifty-nine compounds were identified in the four soup samples, including 22 detected in ESI^+^ and 37 in ESI^−^. Most of the taste-related compounds in chicken soup are related to the free taste compounds present in meat, as well as the degradation of proteins, Maillard reaction, etc. [17]. The 59 compounds were classified into six categories, and the quantitative results are shown in Figure 2. The results showed that the total amount of nucleotides and its metabolites (2.61~8.25 g/kg) were the largest among the six categories of compounds, followed by peptides (0.58~1.04 g/kg) and amino acids and their derivatives (0.42~0.56 g/kg).

#### 3.2.1. Organic Acids

A total of 13 organic acids were detected in the four samples, and the 90 wk soup had the highest (0.46 g/kg) content of organic acids (Figure 2a). Organic acids, an important taste compound in chicken soup, have been detected in many types of chicken [2]. Studies have shown that organic acids are not only derived from the metabolism of sugars, fats, and proteins in chicken, but their exogenous intake (feed, drugs, etc.) was also important [18]. In this work, the total content of organic acids in chicken breast soup was positively correlated with chicken age except in the 20 wk soup. Lactic acid (0.26 g/kg) exhibited the highest content in the 90 wk soup, accounting for 55.53% of the total organic acids content. The content of propionic acid (0.16 g/kg) was next to that of lactic acid. Lactic acid and propionic acid, which have a sour taste, were important for balancing the taste of the soup and are also considered satiety-inducing triggers [19]. The five most abundant compounds (propionic acid, linolenic acid, lactic acid, cinnamic acid, and fumaric acid) were the same in both the 20 and 60 wk soup samples, indicating that the contribution of organic acids to the taste of the chicken breast soup was similar for both ages. The distribution of each compound in the four samples is shown in Figure S1. The results showed that three compounds, including lactic acid, propionic acid, and succinic acid were positively correlated with chicken age, while three compounds, fumaric acid, malic acid, and palmitic acid, were negatively correlated with chicken age. Lactic acid has also been shown to increase with age in ducks aged 50–170 days [20].

#### 3.2.2. Amino Acids and Their Derivatives

A total of 19 amino acids and their derivatives were detected in the four samples, and the 90 wk soup observed the highest (0.56 g/kg) content of amino acids and their derivatives (Figure 2b). Studies have shown that an abundance of amino acids contributes to the formation of taste substances, considering that they are vital compounds in the Maillard reaction [21]. Isoleucine was the most abundant compound in the four samples, and its content percentage in the 90 wk soup sample (40.21%) was the highest. In the study of Liu et al., isoleucine was also considered to be the dominant metabolite in duck meat [20]. Isoleucine has a mildly bitter taste. Studies have shown that it contributes to the sweetness of Chinese rice wine, as well as the sourness of dry-cured ham [22,23]. Therefore, the contribution of isoleucine to the taste of chicken soup needs further clarification. The content of 3-methyl-L-histidine was next to isoleucine in the 10 (0.07 g/kg), 20 (0.07 g/kg), and 90 (0.09 g/kg) wk soup samples. As 3-methyl-L-histidine has not been reported as a taste compound, its contribution to chicken stock is unknown. From Figure 2b, the seven most abundant compounds (isoleucine, 3-methyl-L-histidine, tyrosine, leucine, phenylalanine, 4-aminobutyric acid, and alanine) were the same in both the 10 and 20 wk soup samples, indicating that the contribution of amino acids and their derivatives to the taste of the chicken breast soup was similar for both ages. The distribution of compounds in the four samples showed that the arginine and tyrosine content were negatively correlated with chicken age, while the other compounds did not show a clear pattern with chicken age (Figure S2).

#### 3.2.3. Nucleotides and Their Metabolites

A total of 13 nucleotides and their metabolites were detected in the four samples. The total content of the 13 compounds in the 90 wk soup sample (8.25 g/kg) was the highest (Figure 2c). From Figure 2c, inosine was the most abundant compound in the four samples, and its content percentage in the 90 wk soup sample (35.54%) was the highest. The content of hypoxanthine was next to inosine in the 10 (0.88 g/kg), 20 (0.94 g/kg), and 60 (1.41 g/kg) wk soup samples. Inosine and hypoxanthine, which exhibit a bitter taste, have been found to contribute to the sourness and saltiness of beef semitendinosus muscles [24]. Therefore, the contribution of these two compounds to the taste of chicken breast soup cannot be ignored. The content of 5′-IMP (1.69 g/kg) was next to inosine in the 90 wk soup sample. Studies have shown that 5′-IMP is an important umami compound in muscle, which is derived from the degradation of adenosine triphosphate (ATP) [25]. The content of 5′-IMP decreased and then increased with chicken age and its contents were the lowest in the 60 wk soup sample in this work. Katemala et al. observed that the 5′-IMP of breast meat decreased as Korat hybrid chickens grew older during the 8–20 wk examination, which was consistent with the results observed in the present work [26]. The 5′-IMP levels were significantly higher in 22 wk Hinai-jidori chickens than in 8 wk chickens in the study by Rikimaru et al. [27]. Thus, the relationship between chicken age and 5′-IMP content appears to vary with species and rearing systems. The distribution of the compounds in the four samples showed that the 5′-GMP and uridine content were negatively correlated with chicken age, while the other compounds did not show a clear pattern with chicken age (Figure S3).

#### 3.2.4. Vitamins

A total of 5 vitamins were detected in the four samples, and the 90 wk soup observed the highest (0.113 g/kg) content of vitamins (Figure 2d). Niacinamide was the most abundant compound in the four samples, and its content percentage in the 90 wk soup sample (79.46%) was the highest. Niacinamide was involved in the composition of coenzymes and played an important role in numerous oxidation–reduction reactions [28]. It was reported that dietary supplementation with high doses of nicotinamide (0.10–0.15 g/kg) increased unsaturated fatty acids in chicken breast and improved meat quality by increasing antioxidants, upregulating the expression of myogenic genes, and inhibiting protein ubiquitination [29]. Niacinamide was considered as a marker compound to identify chilled and frozen chicken, and higher niacinamide levels indicated better taste quality in chilled chicken in the study of Wang et al. [30]. The content of nicotinic acid in the four soup samples was 0.007 (10 wk), 0.008 (20 wk), 0.022 (60 wk), and 0.009 (90 wk) g/kg. Nicotinic acid could be converted to nicotinamide in animal liver or other tissues [31]. Liang et al. reported that the content of nicotinic acid in adult hens (130 d) was significantly higher than that of nicotinic acid in old hens (360 d) [32]. However, no significant differences in nicotinic acid content were observed in the chicken soup prepared from the 20 and 60 wk chickens in this work. Carnitine, as a multivitamin, is also an important coenzyme in fat metabolism [33]. The distribution of carnitine in the four samples showed that the carnitine content was generally positively correlated with chicken age, while the other four vitamins did not show a clear pattern with chicken age (Figure S4). In a study on the nutritive profile of goose meat, it was also found that carnitine increased with the increasing age of goose [34]. Nicotinic acid has a sour taste, and nicotinamide is generally known to be bitter [35]. However, little information is available on the contribution of nicotinic acid and niacinamide to the taste of foods.

#### 3.2.5. Peptides

A total of four peptides were detected in the four soup samples, and the 90 wk soup had the highest (1.04 g/kg) content of peptides (Figure 2e). Ala-Phe was the most abundant compound in the 10~60 wk soup samples, while carnosine was the most abundant compound in the 90 wk soup sample. The distribution of the compounds showed that Ala-Phe, carnosine, and anserine were generally positively correlated with chicken age, and their contents were the highest in the 90 wk soup sample (Figure S5). Anserine has also been reported to be positively correlated with age in the early stages of growth in ducks and geese [20,34]. Nishimura et al. reported that carnosine and anserine could buffer the pH of food through their amphoteric groups, thus resulting in a mellow and rich taste of food [36]. Ala-Phe exhibits a bitter taste. Carnosine and anserine contribute to sour and umami tastes, respectively [12]. Glutathione was lower in content than the other three peptides in this work, but its contribution to the taste of the chicken soup should not be missed, given its kokumi taste and its enhancement of umami and saltiness [37].

#### 3.2.6. Other Compounds

The quantitative results of other compounds in the four samples are shown in Figure 2f, and the distribution of each compound in the four samples is shown in Figure S6. The total content of the five other compounds in the 90 wk soup sample (0.52 g/kg) was the highest. Creatinine was the most abundant compound in all four samples, and its content percentage in the 90 wk soup sample (75.66%) was the highest. Creatinine has no taste, but when it reaches twice the natural concentration in meat, it possesses a pronounced bitterness [38]. From Figure S6, the contents of creatinine and ethyl cinnamate were positively correlated with chicken age, and their contents were the highest in the 90 wk soup sample. The contents of furanone, 5-(2-hydroxyethyl)-4-methylthiazole, and 4-hydroxybenzaldehyde decreased and then increased with chicken age.

### 3.3. Pattern Recognition Analysis

**Figure 3:**
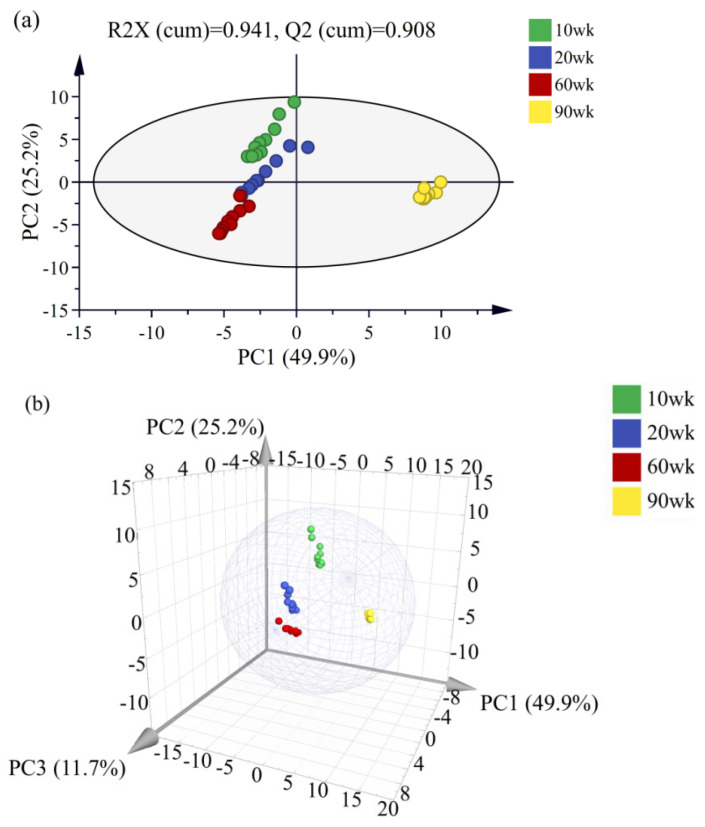
Principal component analysis of four soup samples (2D (**a**) and 3D (**b**) plots).

The results of the hierarchical cluster analysis (HCA) showed that the 10 and 20 wk soup samples were closest and clustered into one group (Group 3), indicating that the difference in compounds between the 10 and 20 wk soup samples was the least (Figure S7). The 60 (Group 2) and 90 wk (Group 1) soup samples were divided into two groups, and the distance between Group 1 and Group 3 was the longest, indicating that there was a great difference in the content of compounds between the old and young chicken soup samples. The principal component analysis (PCA) results of the four soup samples showed that all the samples were distributed within a 95% confidence interval (Figure 3). Additionally, the first three principal components were extracted to explain 86.8% variance information of the characteristic variables of the four soup samples (Figure 3b). From Figure 3a, the 10 and 20 wk soup samples were mainly distributed in the second quadrant, while the 60 and 90 wk soup samples were distributed in the third and the fourth quadrant, respectively. Those results reflected the difference in the compounds between the 90 wk soup sample and the other three samples. This result corresponded to that of the HCA and sensory evaluation.

### 3.4. Differential Compounds Identification

**Figure 4:**
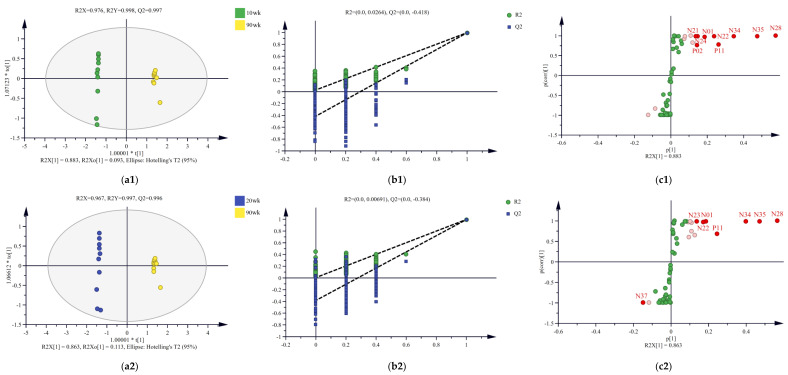

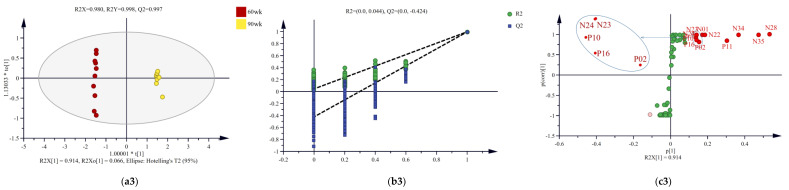
The OPLS-DA scores plots, permutation test plot, and corresponding S-plot of 10/90 (**a1**–**c1**), 20/90 (**a2**–**c2**), and 60/90 (**a3**–**c3**) wk models (**c1**–**c3)**. *, the multiplication sign. The 15 compounds with the largest VIP values in S-plot (**c1**–**c3)** are labeled in light red (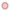), and those with VIP ≥ 1 are labeled in bright red (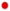). P02, creatinine; P10, isoleucine; P11, hypoxanthine; P16, guanine; N01, lactic acid; N21, propionic acid; N22, carnosine; N23, adenine; N24, anserine; N28, inosine; N34, 5′-AMP; N35, 5′-IMP; N37, glutathione.

OPLS-DA models of the 10/90, 20/90, and 60/90 wk samples were established to investigate the effects of chicken age on the compounds in the chicken breast soup. According to the OPLS-DA score plots (Figure 4(a1–a3)), the three groups of soup were well distinguished, indicating that the age of the chicken had a significant effect on the compounds in the soup. The results of the permutation test (Figure 4(b1–b3)) showed that no overfitting occurred in the three OPLS-DA models, indicating that the obtained results were reliable. Nine, eight, and eleven compounds with VIP ≥ 1 were screened from the 10/90, 20/90, and 60/90 wk models, respectively (Figure 4(c1–c3)). The quantitative results of these compounds (except glutathione in the 20/90 wk model) showed that their levels were higher in the old chicken soup (90 wk) than in the young chicken soup (10, 20, or 60 wk). It was concluded that the upregulated compounds were essential for the development of the taste of the old chicken soup.

The fold change (FC) (FC ≥ 1.2) and *p* (*p* < 0.05) values were used to constrain the compounds according to a previous study [12], and the results are shown in Table 1. Nine differential compounds were identified in the 10/90 wk model, including four nucleotides and their metabolites (5′-AMP, 5′-IMP, inosine, hypoxanthine), two organic acids (lactic acid, propionic acid), two peptides (carnosine, anserine), and one other compound (creatinine). Seven differential compounds were identified in the 20/90 wk model, including five nucleotides and their metabolites (5′-AMP, 5′-IMP, inosine, hypoxanthine, adenine), one organic acid (lactic acid), and one peptide (carnosine). Eleven differential compounds were identified in the 60/90 wk model, including six nucleotides and their metabolites (5′-AMP, 5′-IMP, inosine, hypoxanthine, adenine, guanine), one amino acid (isoleucine), one organic acid (lactic acid), two peptides (carnosine, anserine), and one other compound (creatinine). Six upregulated compounds (carnosine, hypoxanthine, inosine, 5′-IMP, 5′-AMP, and lactic acid) were obtained in all three models, indicating a potential contribution to the taste of the 90 wk soup samples. In general, nucleotides and their metabolites were the main differential compounds in the three models. The results showed that OPLS-DA provided an effective solution to identify differential compounds in soup samples prepared with different ages of hens.

### 3.5. Addition Experiments Results

**Figure 5:**
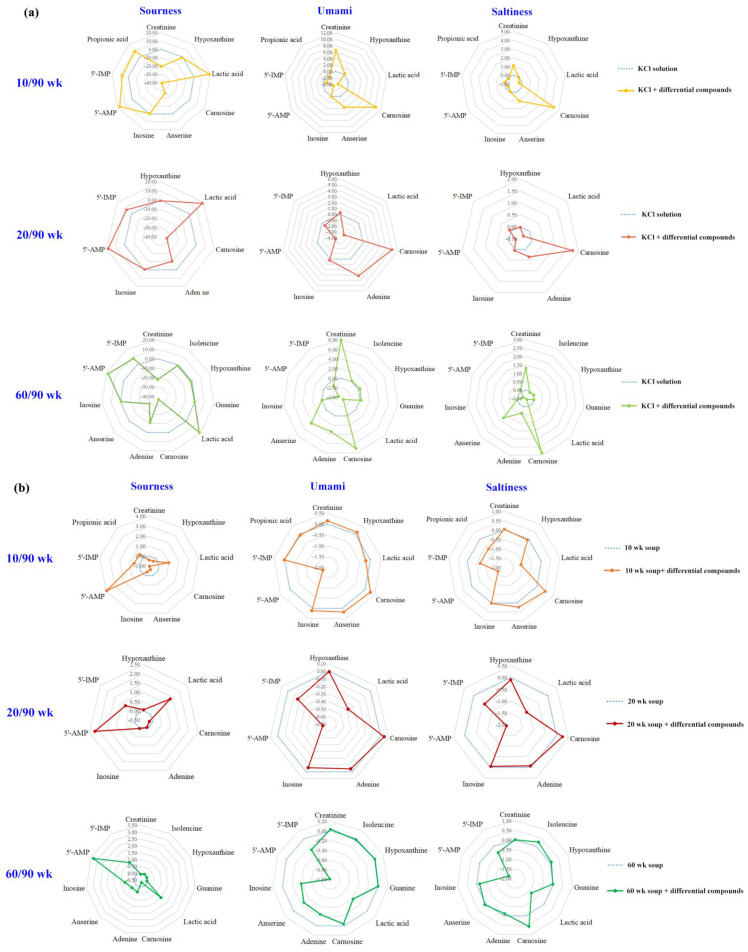
Taste evaluation of differential compounds in KCl solution (**a**) and chicken soup (**b**) by the electronic tongue.

Given the significant effect of chicken age on the sourness, saltiness, and umami of the soup, the contributions of the differential compounds to these three taste attributes of the soup were further studied, and the results are shown in Figure 5. Lactic acid (18.15~18.57), carnosine (5.01~10.28), and carnosine (1.75~4.32) had the highest signal intensity of sourness, umami, and saltiness, respectively (Figure 5a). Among the six compounds common to three models, the signal intensities of lactic acid (sourness), carnosine (umami and saltiness), and 5′-IMP (sourness) were positively correlated with their concentrations. Conversely, the sourness signal intensity of hypoxanthine was negatively correlated with its concentration. A previous study also supports our observations that the compounds had multiple taste properties and the concentration was related to its taste [14].

Additional experiment results showed that lactic acid, 5′-AMP, and 5′-IMP particularly contributed to the sourness of three soup samples (Figure 5b), but no correlation was found between their concentration and the ability to enhance the sourness of the chicken soup. The reason might be the buffering action of the peptides in the chicken soup samples [36]. Compared with lactic acid, which had the highest signal intensity of sourness, 5′-AMP was more efficient at enhancing the sourness of three soup samples. From Figure 5b, the addition of 5′-AMP increased the sourness scores of the 10 (0.49 g/L added), 20 (0.63 g/L added), and 60 (0.64 g/L added) wk soup by 3.91, 2.20, and 3.30 points, respectively, with 5′-AMP increasing the sourness intensity of the 10 wk soup the most. The 5′-AMP was proven to enhance not only the sourness but also the umami taste of stewed sheep tail fat in a previous study [14]. Notably, carnosine without sourness showed an effect of masking the sourness of chicken soup, and its ability to reduce sourness was positively correlated with concentration, which might be due to the amphoteric ions it contains [36]. The umami-enhancing effect of differential compounds was mainly shown in the 10 wk soup samples (Figure 5b). The carnosine with the highest umami intensity had the best umami-enhancing effect in the 10/90 wk model, with the addition of carnosine (0.23 g/L) increasing the umami score of the 10 wk soup by 0.28 points. In a study by Luo et al., carnosine was proven to contribute to the quality and umami taste of meat [39]. Creatinine, anserine, inosine, and hydroxypurine also showed better umami enhancement in this work, of which inosine has been reported to improve the umami and saltiness of stewed sheep tail fat [14]. For saltiness, carnosine and anserine with higher saltiness scores were more effective at enhancing the saltiness of the chicken soup. Among them, the addition of carnosine increased the saltiness scores of the 10 (0.23 g/L added), 20 (0.12 g/L added), and 60 (0.17 g/L added) wk soup by 0.52, 0.26, and 0.57 points, respectively, with carnosine increasing the saltiness intensity of the 60 wk soup the most. Isoleucine without saltiness also showed a saltiness-enhancing effect. Luo et al. reported that isoleucine contributed to the taste of dry-cured mutton ham, and the sourness intensity of ham increased with increasing levels of isoleucine [39]. Considering the synergistic effect of sourness and saltiness, the saltiness enhancement of the chicken soup with isoleucine might be related to its sourness-enhancing ability. Considering the variety of taste-active substances in chicken soup and the complexity of the taste perception in oral processing, the mechanism of taste modulation of differential compounds in chicken soup needs to be further investigated [40].

## 4. Conclusions

In summary, the duration of chicken growth played a significant role in the taste quality of chicken soup. The overall taste score of the soup, as well as the sourness, saltiness, and umami, all increased with the age of chicken. The total content of the 59 identified compounds was the highest in the 90 wk soup sample. The upregulated compounds particularly contributed to the taste profile of the 90 wk soup samples, with carnosine, hypoxanthine, inosine, 5′-IMP, 5′-AMP, and lactic acid being considered the critical potential contributors. Finally, 5′-AMP and carnosine were confirmed as the key taste-active compounds contributing to the taste perception of the 90 wk soup. These results are helpful for taste-quality studies on age profiling in chicken soup and motivating the creation of better chicken soup products which meet the preferences of the consumers.

## Figures and Tables

**Table 1:** The fold change (FC) and *p* value for compounds with VIP ≥ 1 in three models.

10/90 wk			20/90 wk			60/90 wk		
Compound	FC Value	*p* Value	Compound	FC Value	*p* Value	Compound	FC Value	*p* Value
5′-AMP	2.40	<0.01	5′-AMP	3.85	<0.01	5′-AMP	4.04	<0.01
5′-IMP	5.41	<0.01	5′-IMP	4.62	<0.01	5′-IMP	16.54	<0.01
Inosine	3.17	<0.01	Inosine	2.86	<0.01	Inosine	3.19	<0.01
Hypoxanthine	1.70	<0.01	Hypoxanthine	1.58	<0.01	Hypoxanthine	2.21	<0.01
Lactic acid	5.76	<0.01	Adenine	1.45	<0.01	Adenine	1.64	<0.01
Propionic acid	3.60	<0.01	Lactic acid	5.57	<0.01	Guanine	2.32	<0.01
Carnosine	4.71	<0.01	Carnosine	1.70	<0.01	Lactic acid	5.46	<0.01
Anserine	2.07	<0.01	Glutathione	0	<0.01	Carnosine	2.35	<0.01
Creatinine	1.61	<0.01				Anserine	2.17	<0.01
						Creatinine	2.10	<0.01
						Isoleucine	2.87	<0.01

## Data Availability

Data is contained within the article.

## Supplementary Materials

The following supporting information can be downloaded at: https://www.mdpi.com/article/10.3390/foods12030674/s1, Figure S1: The distribution of organic acids (a-m) in four soup samples (** *p* < 0.01, * *p* < 0.05); Figure S2: The distribution of amino acids and their derivatives (a-s) in four soup samples (** *p* < 0.01, * *p* < 0.05); Figure S3: The distribution of nucleotides and their metabolites (a-m) in four soup samples (** *p* < 0.01, * *p* < 0.05); Figure S4: The distribution of vitamins (a-e) in four soup samples (** *p* < 0.01, * *p* < 0.05); Figure S5: The distribution of peptides (a-d) in four soup samples (** *p* < 0.01, * *p* < 0.05); Figure S6: The distribution of other compounds (a-e) in four soup samples (** *p* < 0.01, * *p* < 0.05); Figure S7: The hierarchical clustering analysis (HCA) of four soup samples (The greater distance between samples indicates greater variation.); Table S1: Basic ingredients of four chicken breast samples; Table S2: Chemicals information; Table S3: Instrument conditions for LC-QTOF-MS/MS analysis; Table S4: Kruskal-Wallis test results for taste profiles of four chicken soup samples; Table S5: Compounds identified in four soup samples.
