# Supplementary material for: Decoding the Effect of Age on the Taste Perception of Chicken Breast Soup Based on LC-QTOF-MS/MS Combined with a Chemometric Approach
Source: Foods. 2023 Feb 3;12(3):674. doi: 10.3390/foods12030674 (PMC9914887; doi:10.3390/foods12030674)
Supplement: Supplementary file 1 [file foods-12-00674-s001.zip › foods-2126321-supplementary.pdf]

Table S1. Basic ingredients of four chicken breast samples.

Sample	Basic components (%)				
	Moisture	Ash	Crude protein	Crude fat	Glucose
10 wk chicken breast	75.6	1.0	23.0	0.8	0.5
20 wk chicken breast	74.2	1.1	23.8	1.4	0.5
60 wk chicken breast	72.5	1.0	24.7	1.7	0.31
90 wk chicken breast	72.8	1.4	24.6	2.9	0.5

Table S2. Chemicals information.

Chemicals	Purity	Manufacturers
Formic acid	98%	Bailingwei (Beijing, China)
Malic acid	99%	Bailingwei (Beijing, China)
Adenosine 5'-diphosphate	98%	Bailingwei (Beijing, China)
Nicotinic acid	99.5%	Bailingwei (Beijing, China)
Choline hydroxide	47.3%	Bailingwei (Beijing, China)
Inosine	98%	Aladdin (Shanghai, China)
Carnosine	97%	Aladdin (Shanghai, China)
Isoleucine	99%	Aladdin (Shanghai, China)
Creatinine	99%	Aladdin (Shanghai, China)
Proline	99%	Aladdin (Shanghai, China)
Aspartic acid	99%	Aladdin (Shanghai, China)
Glutamic acid	99%	Aladdin (Shanghai, China)
Histidine	99%	Aladdin (Shanghai, China)
Phenylalanine	99%	Aladdin (Shanghai, China)
Tyrosine	99%	Aladdin (Shanghai, China)
Methionine	99%	Aladdin (Shanghai, China)
3-Methyl-L-histidine	98%	Aladdin (Shanghai, China)
Arginine	98%	Aladdin (Shanghai, China)
Valine	99%	Aladdin (Shanghai, China)
Alanine	99%	Aladdin (Shanghai, China)
4-Aminobutyric acid	99%	Aladdin (Shanghai, China)
Guanosine	98%	Aladdin (Shanghai, China)
Glutathione	99%	Aladdin (Shanghai, China)
4-Hydroxybenzaldehyde	99%	Aladdin (Shanghai, China)
Leucine	99%	Aladdin (Shanghai, China)
Nicotinamide	99.5%	Aladdin (Shanghai, China)
Theanine	99%	Aladdin (Shanghai, China)
Tryptophan	99%	Aladdin (Shanghai, China)
Hypoxanthine	99%	Macklin (Shanghai, China)
Guanine	99%	Macklin (Shanghai, China)
Anserine	97%	Macklin (Shanghai, China)
Adenine	99.5%	Macklin (Shanghai, China)
Pyroglutamic acid	99%	Macklin (Shanghai, China)

Table S2. Continued.

Chemicals	Purity	Manufacturers
Adenosine	99.5%	Macklin (Shanghai, China)
Lactic acid	98%	Jinming Bio-Technology (Beijing, China)
Inosine 5'-monophosphate	98%	Sigma-Aldrich (Shanghai, China)
Adenosine 5'-monophosphate	97%	Sigma-Aldrich (Shanghai, China)
Disodium 5'-Guanylate	99%	Sigma-Aldrich (Shanghai, China)
Taurine	99%	Sigma-Aldrich (Shanghai, China)
5-(2-Hydroxyethyl)-4-methylthiazole	98%	Sigma-Aldrich (Shanghai, China)
Ethyl cinnamate	98%	Sigma-Aldrich (Shanghai, China)
Palmitic acid	99%	Sigma-Aldrich (Shanghai, China)
Propionic acid	99%	TCI (Shanghai, China)
Tartaric acid	99%	Sinopharm (Shanghai, China)
Succinic acid	99.5%	Sinopharm (Shanghai, China)
Fumaric acid	99%	Sinopharm (Shanghai, China)
Citric acid	99.8%	Sinopharm (Shanghai, China)
Betaine	99%	Yuanye Bio-Technology (Shanghai, China)
Cinnamic acid	99.5%	Yuanye Bio-Technology (Shanghai, China)
2,6-Dihydroxypurine	98%	Yuanye Bio-Technology (Shanghai, China)
Uric acid	99%	Yuanye Bio-Technology (Shanghai, China)
Kojic acid	99%	Yuanye Bio-Technology (Shanghai, China)
Carnitine	98%	Yuanye Bio-Technology (Shanghai, China)
Uridine	99%	Yuanye Bio-Technology (Shanghai, China)
Riboflavin	98%	Yuanye Bio-Technology (Shanghai, China)
Cholic acid	97%	Yuanye Bio-Technology (Shanghai, China)
Furaneol	98%	Yuanye Bio-Technology (Shanghai, China)
Linolenic acid	98%	Yuanye Bio-Technology (Shanghai, China)
Linoleic acid	98%	Yuanye Bio-Technology (Shanghai, China)

Table S3. Instrument conditions for LC-QTOF-MS/MS analysis.

**Gradient elution procedure**		
Time (min)	Proportion of mobile phase A(C) (%)	
0	95	
33	82	
46	65	
49	20	
53	20	
54	5	
60	95	
**Mass spectrum parameters**		
	ESI^+ mode	ESI^- mode
Scan range for MS and MS/MS	50~1000 Da	50~1000 Da
Capillary voltage	+5500 V	-4500 V
Gas 1	55 psi	55 psi
Gas 2	60 psi	60 psi
Curtain gas	35 psi	35 psi
Electrospray ion source temperature	550 °C	550 °C
Declustering potential	+80V	-80V
Collision energy	+35±15V	-35±15V

Table S4. Kruskal–Wallis test results for taste profiles of four chicken soup samples.

	10 wk	20 wk	60 wk	90 wk	p^a
Sourness	2.00 (1.00, 2.25)	2.00 (1.75, 3.25)	3.00 (2.75, 3.25)	4.00 (2.75, 4.00)	0.003**
Sweetness	2.00 (1.00, 2.25)	2.00 (1.00, 2.25)	1.00 (1.00, 1.25)	1.00 (1.00, 2.25)	0.212
Bitterness	1.00 (1.00, 1.25)	2.00 (1.00, 2.00)	1.00 (1.00, 1.25)	2.00 (1.00, 2.00)	0.090
Saltiness	2.00 (2.00, 2.00)	3.00 (2.00, 3.25)	3.00 (2.75, 4.25)	4.00 (3.00, 4.50)	<0.001**
Umami	6.00 (4.75, 6.00)	5.00 (4.00, 7.00)	7.00 (5.25, 7.00)	8.00 (8.00, 8.00)	<0.001**
Overall taste	5.00 (4.00, 5.00)	5.00 (4.75, 7.00)	5.00 (4.00, 6.00)	6.00 (5.00, 6.25)	0.031*

The results are reported as median (25th percentile (P25), 75th percentile (P75)). [a] Kruskal–Wallis test was used to compare variables between the groups. **$p<0.01$, *$p<0.05$.

Table S5. Compounds identified in four soup samples.

No.	Compound	Molecular weight	Mode	No.	Compound	Molecular weight	Mode
P01	Adenosine	267.24	ESI^+	N09	Aspartic acid	133.1	ESI^-
P02	Creatinine	113.12	ESI^+	N10	Malic acid	134.09	ESI^-
P03	Proline	115.13	ESI^+	N11	Glutamic acid	147.13	ESI^-
P04	Betaine	117.15	ESI^+	N12	Cinnamic acid	148.16	ESI^-
P05	5'-GMP	387.22	ESI^+	N13	2,6-Dihydroxypurine	152.11	ESI^-
P06	Choline	121.18	ESI^+	N14	Histidine	155.15	ESI^-
P07	Nicotinamide	122.12	ESI^+	N15	Phenprobamate	165.19	ESI^-
P08	Nicotinic acid	123.11	ESI^+	N16	Uric acid	168.11	ESI^-
P09	Furaneol	128.13	ESI^+	N17	Theanine	174.2	ESI^-
P10	Isoleucine	131.17	ESI^+	N18	Tyrosine	181.19	ESI^-
P11	Hypoxanthine	136.11	ESI^+	N19	Citric acid	192.12	ESI^-
P12	Kojic acid	142.11	ESI^+	N20	Tryptophan	204.23	ESI^-
P13	5-(2-Hydroxyethyl)-4-methylthiazole	143.21	ESI^+	N21	Propionic acid	74.08	ESI^-
P14	Ala-Phe	236.27	ESI^+	N22	Carnosine	226.23	ESI^-
P15	Methionine	149.21	ESI^+	N23	Adenine	135.13	ESI^-
P16	Guanine	151.13	ESI^+	N24	Anserine	240.26	ESI^-
P17	Carnitine	161.2	ESI^+	N25	Uridine	244.2	ESI^-
P18	Ethyl cinnamate	162.19	ESI^+	N26	Palmitic acid	256.42	ESI^-
P19	3-Mehtyl-L-Histidine	169.18	ESI^+	N27	Tartaric acid	150.09	ESI^-
P20	Arginine	174.2	ESI^+	N28	Inosine	268.23	ESI^-
P21	Riboflavin	376.36	ESI^+	N29	Linolenic acid	278.43	ESI^-
P22	Adenosine 5'-diphosphate	427.2	ESI^+	N30	Linoleic acid	280.45	ESI^-
N01	Lactic acid	90.08	ESI^-	N31	Valine	117.15	ESI^-
N02	4-Aminobutyric acid	103.12	ESI^-	N32	Alanine	89.09	ESI^-
N03	Fumaric acid	116.07	ESI^-	N33	Guanosine	283.24	ESI^-
N04	Succinic acid	118.09	ESI^-	N34	5'-AMP	347.22	ESI^-
N05	4-Hydroxybenzaldehyde	122.12	ESI^-	N35	5'-IMP	348.21	ESI^-
N06	Taurine	125.15	ESI^-	N36	Cholic acid	408.58	ESI^-
N07	Pyroglutamic acid	129.11	ESI^-	N37	Glutathione	612.63	ESI^-
N08	Leucine	131.17	ESI^-				

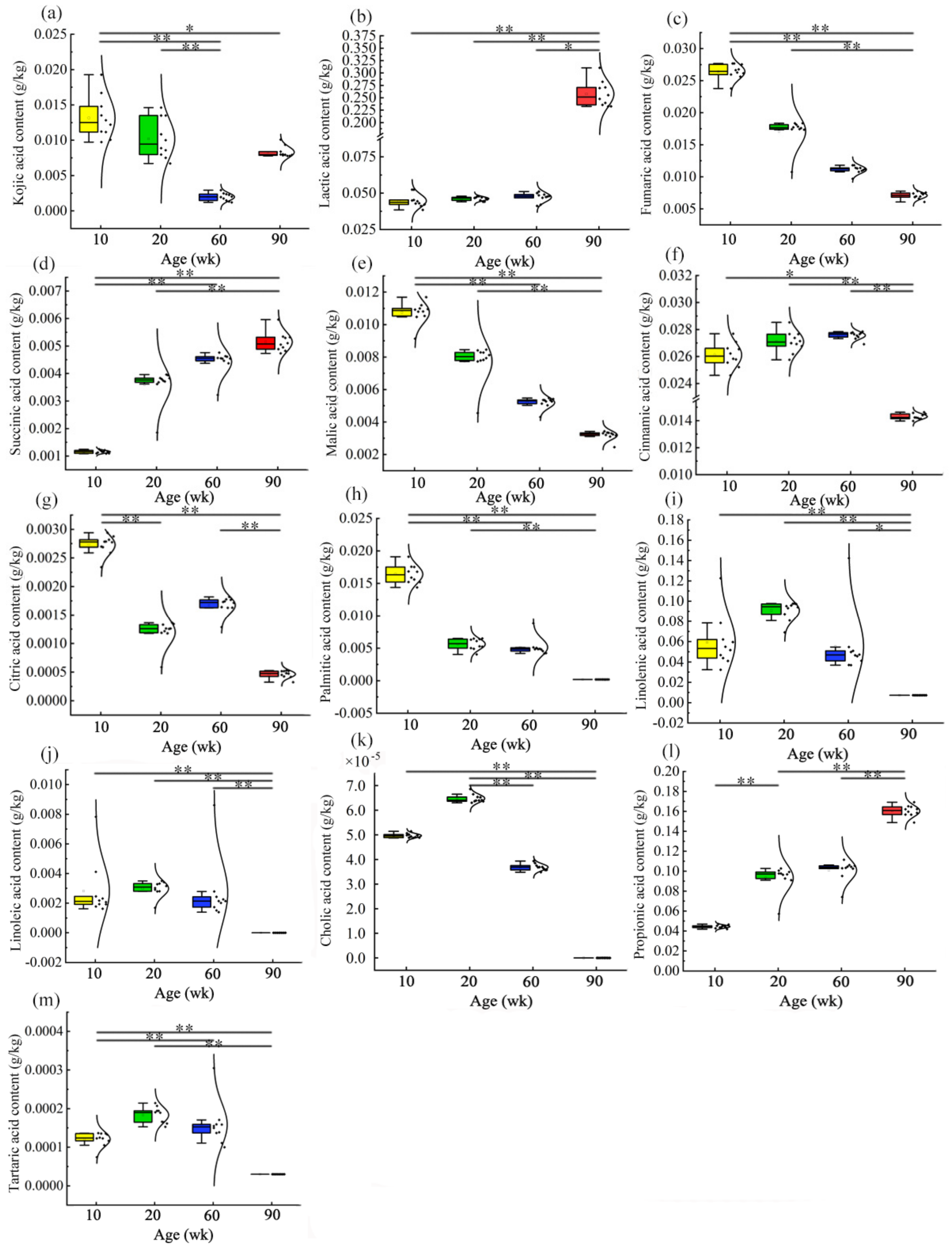

Figure S1. The distribution of organic acids (a-m) in four soup samples (**$p<0.01$, *$p<0.05$).

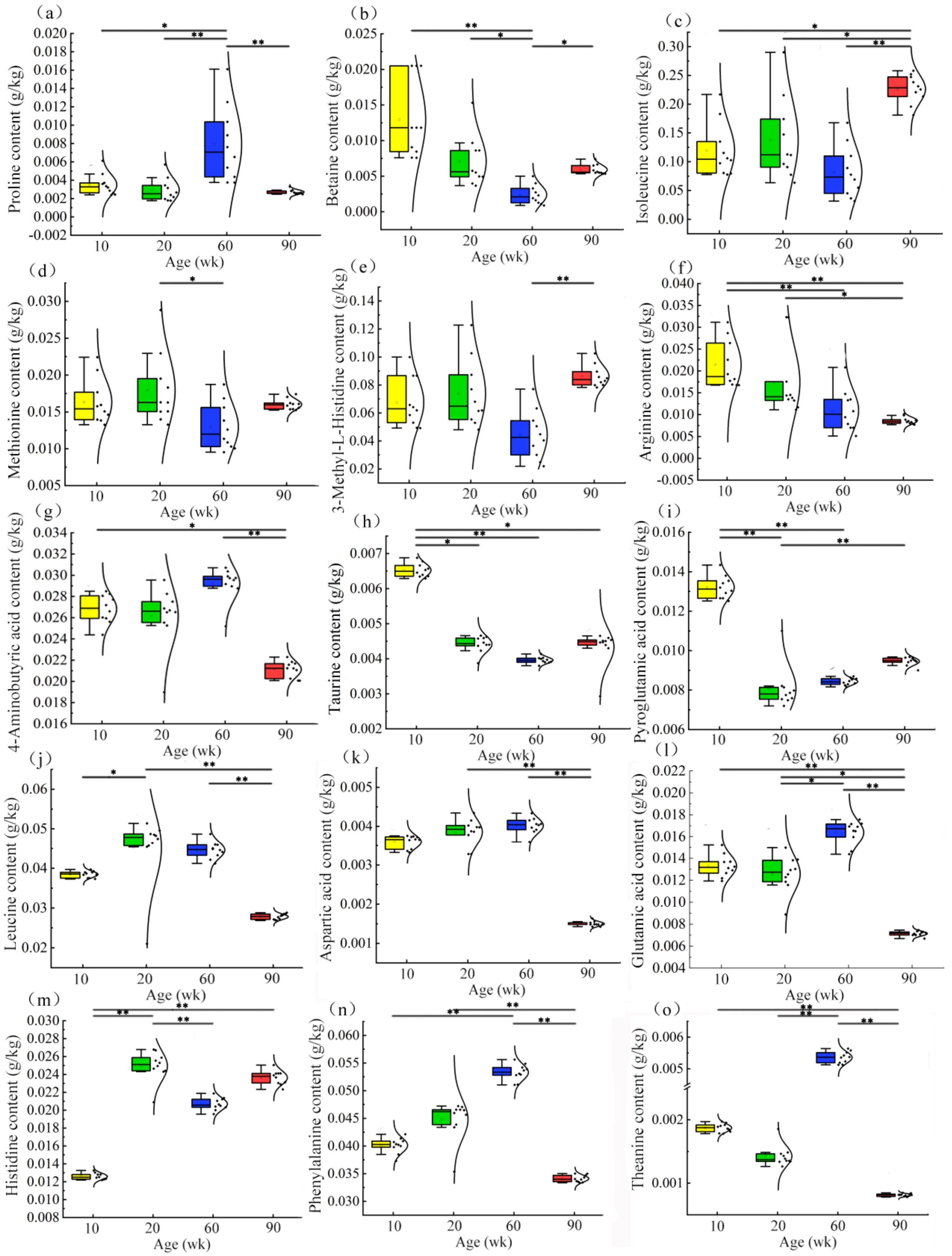
(a)
Proline content (g/kg)
Age (wk)
(b)
Betaine content (g/kg)
Age (wk)
(c)
Isoleucine content (g/kg)
Age (wk)
(d)
Methionine content (g/kg)
Age (wk)
(e)
3-Methyl-L-Histidine content (g/kg)
Age (wk)
(f)
Arginine content (g/kg)
Age (wk)
(g)
4-Aminobutyric acid content (g/kg)
Age (wk)
(h)
Taurine content (g/kg)
Age (wk)
(i)
Pyroglutamic acid content (g/kg)
Age (wk)
(j)
Leucine content (g/kg)
Age (wk)
(k)
Aspartic acid content (g/kg)
Age (wk)
(l)
Glutamic acid content (g/kg)
Age (wk)
(m)
Histidine content (g/kg)
Age (wk)
(n)
Phenylalanine content (g/kg)
Age (wk)
(o)
Theanine content (g/kg)
Age (wk)
10
20
60
90

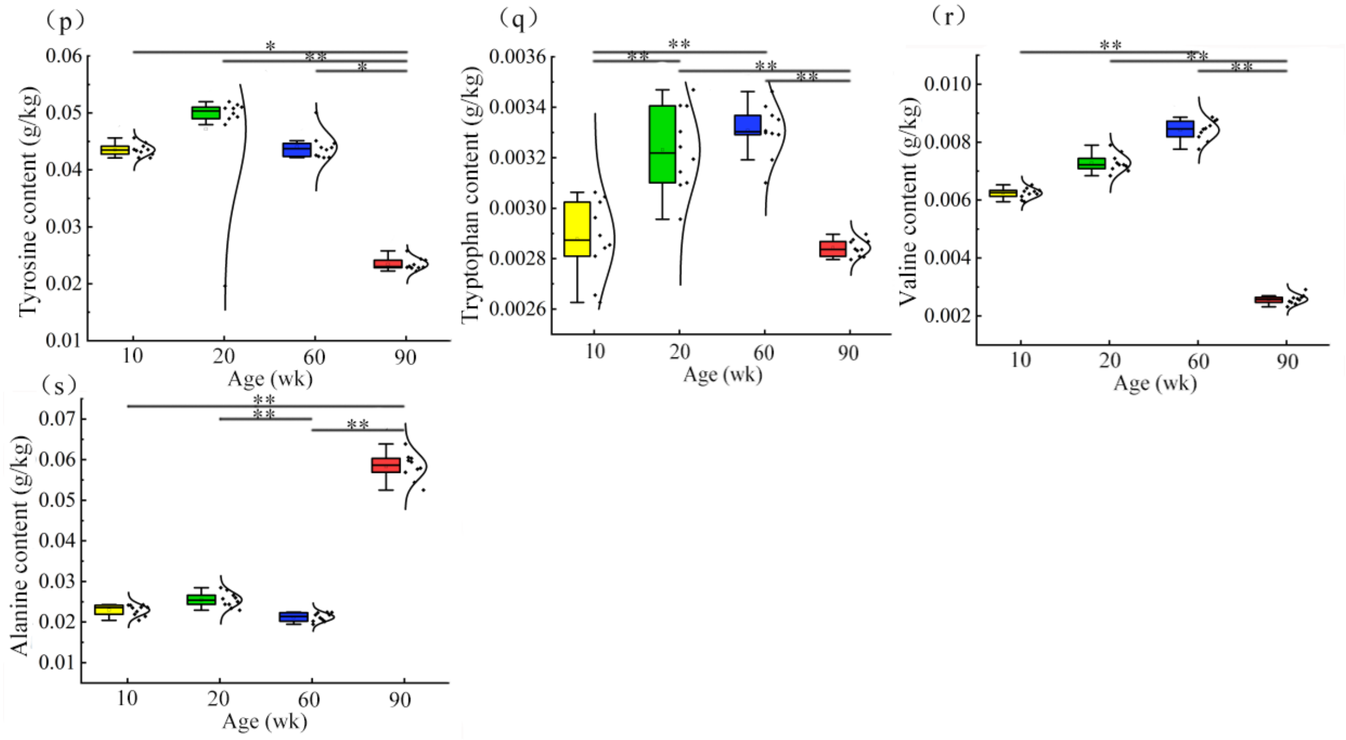

Figure S2. The distribution of amino acids and their derivatives (a-s) in four soup samples (**$p < 0.01$, *$p < 0.05$).

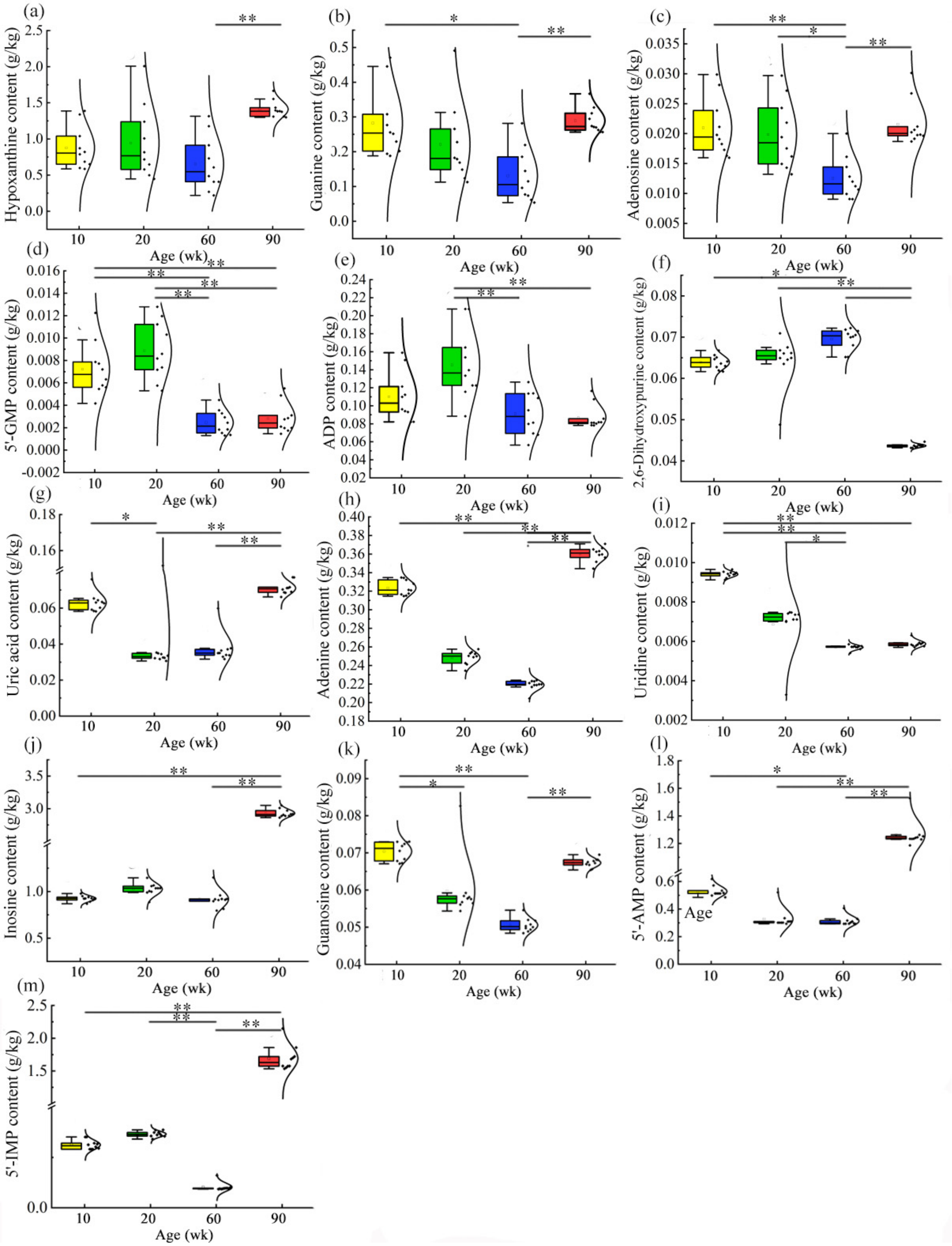

Figure S3. The distribution of nucleotides and their metabolites (a-m) in four soup samples (**p< 0.01, *p<0.05).

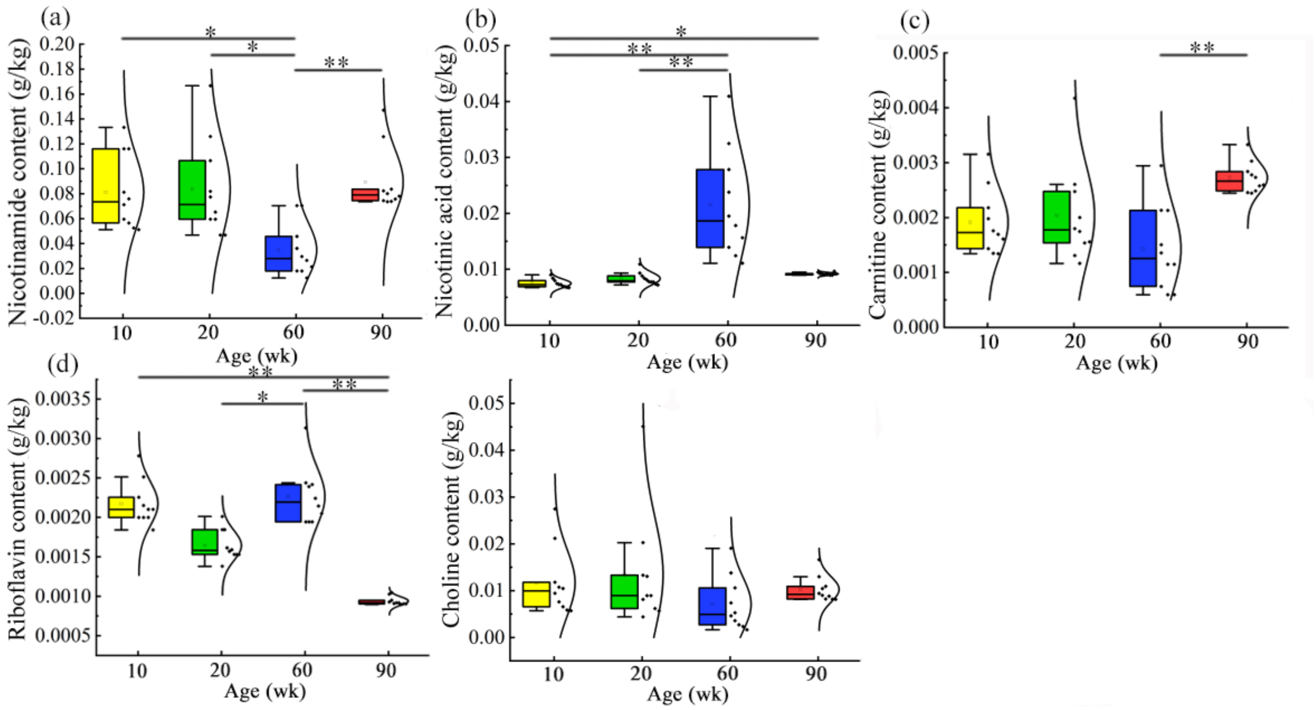

Figure S4. The distribution of vitamins (a-e) in four soup samples (**$p<0.01$, *$p<0.05$).

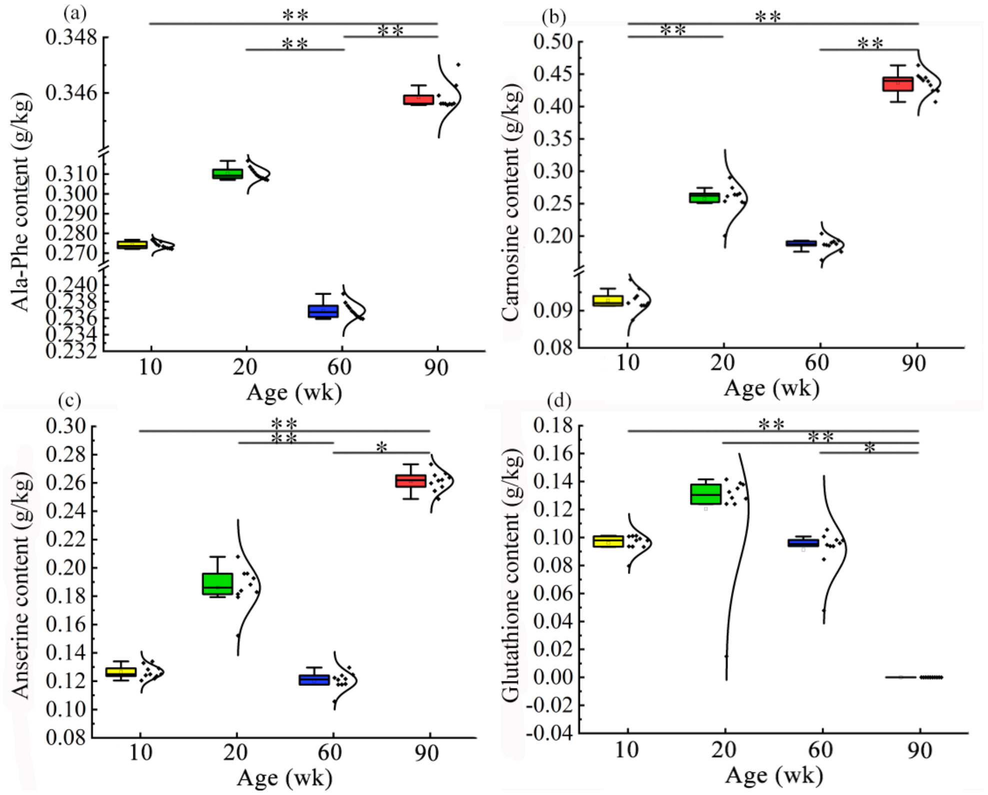

Figure S5. The distribution of peptides (a-d) in four soup samples (**$p<0.01$, *$p<0.05$).

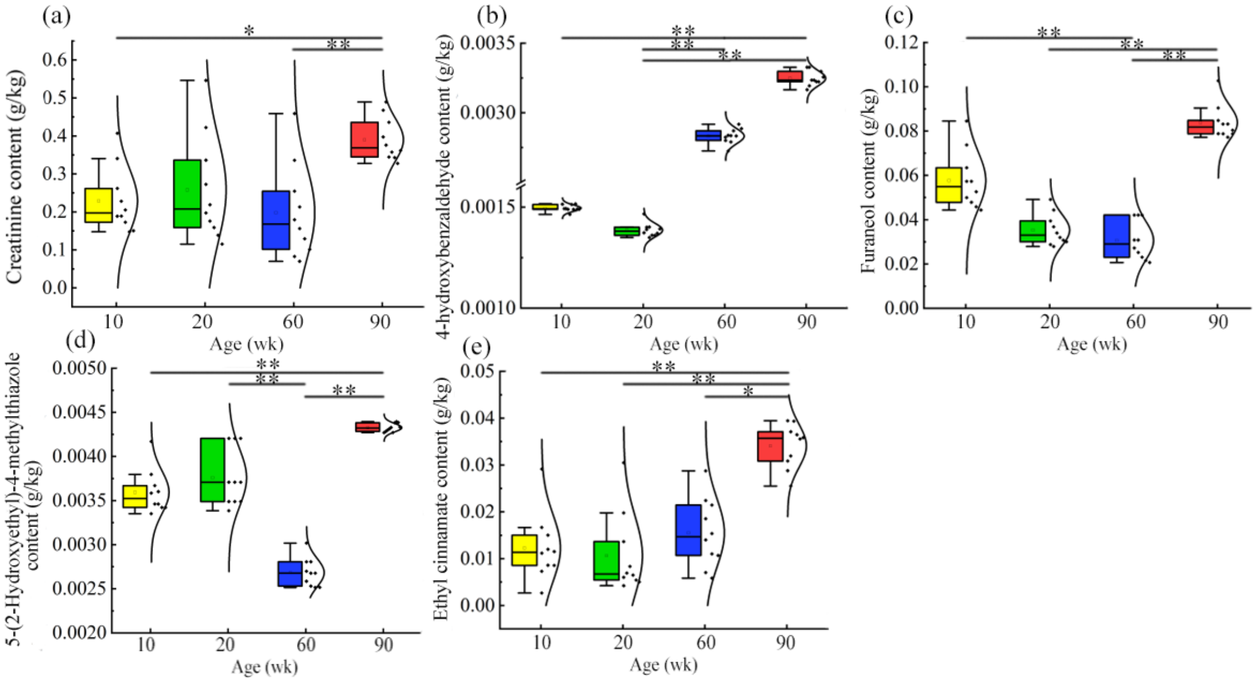

Figure S6. The distribution of other compounds (a-e) in four soup samples (**$p<0.01$, *$p<0.05$).

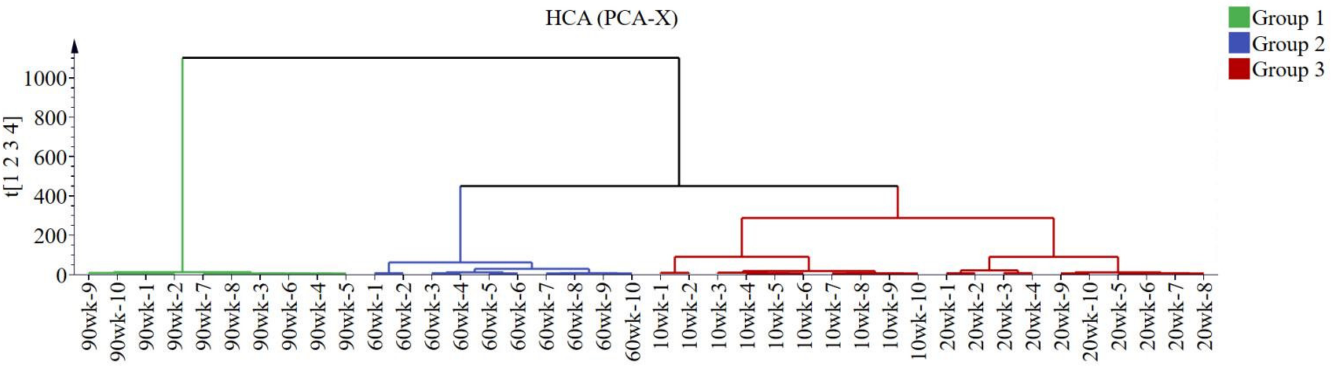

Figure S7. The hierarchical clustering analysis (HCA) of four soup samples (the greater distance between samples indicates greater variation).
